# NutriNet: A Deep Learning Food and Drink Image Recognition System for Dietary Assessment

**DOI:** 10.3390/nu9070657

**Published:** 2017-06-27

**Authors:** Simon Mezgec, Barbara Koroušić Seljak

**Affiliations:** 1 Information and Communication Technologies, Jožef Stefan International Postgraduate School, Jamova Cesta 39, 1000 Ljubljana, Slovenia; 2 Computer Systems Department, Jožef Stefan Institute, Jamova Cesta 39, 1000 Ljubljana, Slovenia; barbara.korousic@ijs.si

**Keywords:** NutriNet, deep convolutional neural networks, deep learning, food recognition, food detection, drink recognition, drink detection, Parkinson’s disease

## Abstract

Automatic food image recognition systems are alleviating the process of food-intake estimation and dietary assessment. However, due to the nature of food images, their recognition is a particularly challenging task, which is why traditional approaches in the field have achieved a low classification accuracy. Deep neural networks have outperformed such solutions, and we present a novel approach to the problem of food and drink image detection and recognition that uses a newly-defined deep convolutional neural network architecture, called NutriNet. This architecture was tuned on a recognition dataset containing 225,953 512 × 512 pixel images of 520 different food and drink items from a broad spectrum of food groups, on which we achieved a classification accuracy of 86.72%, along with an accuracy of 94.47% on a detection dataset containing 130,517 images. We also performed a real-world test on a dataset of self-acquired images, combined with images from Parkinson’s disease patients, all taken using a smartphone camera, achieving a top-five accuracy of 55%, which is an encouraging result for real-world images. Additionally, we tested NutriNet on the University of Milano-Bicocca 2016 (UNIMIB2016) food image dataset, on which we improved upon the provided baseline recognition result. An online training component was implemented to continually fine-tune the food and drink recognition model on new images. The model is being used in practice as part of a mobile app for the dietary assessment of Parkinson’s disease patients.

## 1. Introduction

As people are becoming increasingly aware of the importance of a healthy diet, the need for automatic food and drink recognition systems has arisen. Not only can such systems provide the automatic recognition of food and drink items, but they can also enable an estimation of their nutritional values, making them especially useful for dietary assessment and planning, which is applicable for patients with different dietary restrictions, as well as for healthy individuals by preventing nutrition-related conditions.

The problem of food and drink image detection and recognition is challenging due to the nature of food and drink items. Foods are typically deformable objects, which makes the process of defining their structure difficult. Furthermore, some food types can have a high intra-class (similar foods look very different) and low inter-class (different foods look very similar) variance, making the process of specifying the food type even more challenging. The issue with drink recognition is that there is only a limited amount of information that can be gained using images of drink items; an example of such information is the drink’s color, whether the drink is well-lit, and the drink’s density. All of these obstacles make food and drink image detection and recognition a particularly challenging computer vision problem.

We approached this problem using deep learning or deep neural networks [1]. Many problems in computer vision require the definition of complex features that are very challenging and time consuming to manually define. Deep learning alleviates this as it allows computational models composed of multiple processing layers to automatically learn these features and represent the input data with them. Deep learning models have substantially improved the best results in a variety of research fields, computer vision being one of them [1]. Specifically, we are using deep convolutional neural networks, which are a type of deep neural network that is inspired by the visual cortex of animals, where the individual neurons react to overlapping regions in the visual field [2]. This makes convolutional neural networks especially suitable for computer vision, as the goal of computer vision systems is the same as that of animal vision systems: to gain an understanding of input images.

**Figure 1:**
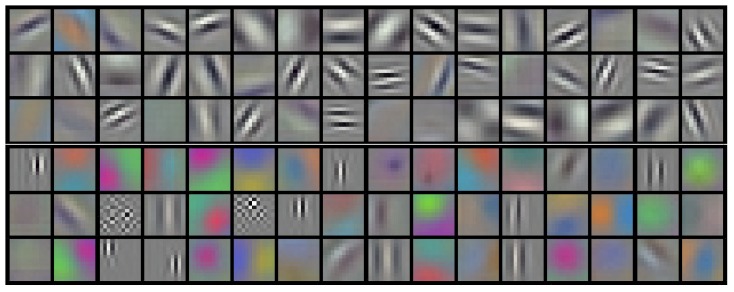
Example filters by Krizhevsky et al. [3]. Because these filters were learned using the first convolutional layer of the neural network, the represented features are simple, such as the edge orientation and frequency (learned features become progressively more complex with each additional convolutional layer). Reproduced with permission from Alex Krizhevsky, Advances in NIPS 25; published by Curran Associates, Inc., 2012.

When an image is fed through a convolutional neural network, a series of operations is performed on the image as data transitions through the network layers. The layer parameters are then adjusted in each iteration, which is how the training is performed. Three types of layers are the most common in convolutional neural networks: convolutional, fully-connected and pooling layers. Convolutional layers contain learnable filters that are trained in such a way that they respond to certain features in the input data; an example of learned filters is shown in Figure 1. Fully-connected layers, on the other hand, compose output data from other layers to gain higher-level knowledge from it. The pooling layers down-sample the input data, but since this layer type does not accept parameters, it is usually not counted towards the total neural network layer depth.

The structure of this paper is as follows. In Section 1.1, related work in the field of food image detection and recognition is presented; in Section 2.1, our image datasets and their acquisition are described; Section 2.2 contains information about NutriNet and other convolutional neural network models that were tested, along with their training process; Section 2.3 describes how the online training component was implemented; in Section 3, the training and testing results of the deep neural network models are given; in Section 3.1, we present testing results of the NutriNet architecture on other datasets, including a real-world image dataset that we built for this purpose; Section 4 contains the discussion part of the research work; and Section 5 concludes the paper and gives an overview of the work done, as well as possible future work.

### 1.1. Related Work

While there have not been any dedicated drink image recognition systems, there have been multiple approaches to food image recognition in the past, and we will briefly mention the most important ones here. In 2009, an extensive food image and video dataset was built to encourage further research in the field: the Pittsburgh Fast-Food Image Dataset (PFID), containing 4545 still images, 606 stereo image pairs, 303 360° food videos and 27 eating videos of 101 different food items, such as “chicken nuggets” and “cheese pizza” [4]. Unfortunately, this dataset focuses only on fast-food items, not on foods in general. The authors provided the results of two baseline recognition methods tested on the PFID dataset, both using an SVM (Support Vector Machine) classifier to differentiate between the learned features; they achieved a classification accuracy of 11% with the color histogram method and 24% with the bag-of-SIFT-features method. The latter method counts the occurrences of local image features described by the popular SIFT (Scale-Invariant Feature Transform) descriptor [5]. These two methods were chosen based on their popularity in computer vision applications, but the low classification accuracy showed that food image recognition is a challenging computer vision task, requiring a more complex feature representation.

In the same year, a food image recognition system that uses the multiple kernel learning method was introduced, which tested different feature extractors, and their combination, on a self-acquired dataset [6]. This proved to be a step in the right direction, as the authors achieved an accuracy of 26% to 38% for the individual features they used and an accuracy of 61.34% when these features were combined; the features include color, texture and SIFT information. Upon conducting a real-world test on 166 food images taken with mobile phones, the authors reported a lower classification accuracy of 37.35%, which was due to factors like occlusion, noise and additional items being present in the real-world images. The fact that the combination of features performed better than the individual features further hinted at the need for a more in-depth representation of the food images. Next year, the pairwise local features method, which applies the specifics of food images to their recognition, was presented [7]. This method analyzes the ingredient relations in the food image, such as the relations between bread and meat in a sandwich, by computing pairwise statistics between the local features. The authors performed an evaluation of their algorithm on the PFID dataset and achieved an accuracy of 19% to 28%, depending on which measure they employed in the pairwise local features method. However, they also noted that the dataset had narrowly-defined food classes, and after joining them into 7 classes, they reported an accuracy of 69% to 78%. This further confirmed the limitations of food image recognition approaches of that time: if a food image recognition algorithm achieved a high classification accuracy, it was only because the food classes were very general (e.g., “chicken”).

In 2014, another approach was presented that uses an optimized bag-of-features model for food image recognition [8]. The authors tested 14 different color and texture descriptors for this model and found that the HSV-SIFT descriptor provided the best result. This descriptor describes the local textures in all three color channels of the HSV color space. The model was tested on a food image dataset that was built for the aims of the project Type 1 Diabetes Self-Management and Carbohydrate Counting: A Computer Vision Based Approach (GoCARB) [9], in the scope of which they constructed a food recognition system for diabetes patients. The authors achieved an accuracy of 77.80%, which was considerably higher than previous approaches.

All of the previously-described solutions are based on manually-defined feature extractors that rely on specific features, such as color or texture, to recognize the entire range of food images. Furthermore, the images used in the recognition systems presented in these solutions were taken under strict conditions, containing only one food dish per image and often perfectly cropped. The images that contained multiple items were manually segmented and annotated, so the final inputs for these hand-crafted recognition systems were always ideally-prepared images. The results from these research works are therefore not indicative of general real-world performance due to the same problems with real-world images as listed above.

These issues show that hand-crafted approaches are not ideal for a task as complex as food image recognition, where it seems the best approach is to use a complex combination of a large number of features, which is why deep convolutional neural networks, a method that automatically learns appropriate image features, achieved the best results in the field. Deep neural networks can also learn to disregard surrounding noise with sufficient training data, eliminating the need for perfect image cropping. Another approach for the image segmentation task is to train a neural network that performs semantic segmentation, which directly assigns class labels to each region of the input image [10,11]. Furthermore, deep neural networks can be trained in such a way that they perform both object detection and recognition in the same network [12,13].

In 2014, Kawano et al. used deep convolutional neural networks to complement hand-crafted image features [14] and achieved a 72.26% accuracy on the University of Electro-Communications Food 100 (UEC-FOOD100) dataset that was made publicly available in 2012 [15]; this was the highest accuracy on the dataset at that time. Also in 2014, a larger version of the UEC-FOOD100 dataset was introduced, the University of Electro-Communications Food 256 (UEC-FOOD256), which contains 256 as opposed to 100 food classes [16]; while UEC-FOOD100 is composed of mostly Japanese food dishes, UEC-FOOD256 expands on this dataset with some international dishes. At that time, another food image dataset was made publicly available: the Food-101 dataset. This dataset contains 101,000 images of 101 different food items, and the authors used the popular random forest method for the recognition task, with which they achieved an accuracy of 50.76% [17]. They reported that while this result outperformed other hand-crafted efforts, it could not match the accuracy that deep learning approaches provided. This was further confirmed by the subsequently published research works, such as by Kagaya et al., who tested both food detection and food recognition using deep convolutional neural networks on a self-acquired dataset and achieved encouraging results: a classification accuracy of 73.70% for the recognition and 93.80% for the detection task [18]. In 2015, Yanai et al. improved on the best UEC-FOOD100 result, again with deep convolutional neural networks, only this time, with pre-training on the ImageNet dataset [19]. The accuracy they achieved was 78.77% [20]. A few months later, Christodoulidis et al. presented their own food recognition system that uses deep convolutional neural networks, and with it, they achieved an accuracy of 84.90% on a self-acquired and manually-annotated dataset [21].

In 2016, Singla et al. used the famous GoogLeNet deep learning architecture [22], which is described in Section 2.2, on two datasets of food images, collected using cameras and combined with images from existing image datasets and social media. With a pre-trained model, they reported a recognition accuracy of 83.60% and a detection accuracy of 99.20% [23]. Also in 2016, Liu et al. achieved similarly encouraging results on the UEC-FOOD100, UEC-FOOD256 and Food-101 datasets by using an optimized convolution technique in their neural network architecture [24], which allowed them to reach an accuracy of 76.30%, 54.70% and 77.40%, respectively. Furthermore, Tanno et al. introduced DeepFoodCam, which is a smartphone food image recognition application that uses deep convolutional neural networks with a focus on recognition speed [25]. Another food image dataset was made publicly available in that year: the University of Milano-Bicocca 2016 (UNIMIB2016) dataset [26]. This dataset is composed of images of 1027 food trays from an Italian canteen, containing a total of 3616 food instances, divided into 73 food classes. The authors tested a combined segmentation and recognition deep convolutional neural network model on this dataset and achieved an accuracy of 78.30%. Finally, in 2016, Hassannejad et al. achieved the current best classification accuracy values of 81.45% on the UEC-FOOD100 dataset, 76.17% on the UEC-FOOD256 dataset and 88.28% on the Food-101 dataset [27]. All three results were obtained by using a deep neural network model based on the Google architecture Inception; this architecture is the basis for the previously-mentioned GoogLeNet.

It seems that deep learning is a very promising approach in the field of food image recognition. Previous deep learning research reported high classification accuracy values, thus confirming the viability of the approach, but they focused on smaller food image datasets, often limited to 100 different food items or less. Moreover, none of these solutions recognize drink images. In this paper, we will present our solution that addresses these issues. We developed a new deep convolutional neural network architecture called NutriNet and trained it on images acquired from web searches for individual food and drink items. With this architecture, we achieved a higher classification accuracy than most of the results presented above and found that, on our recognition dataset, it performs better than AlexNet, which is the deep learning architecture it is based on; the results are described in-depth in Section 3. Additionally, we developed an online training component that automatically fine-tunes the deep learning image recognition model upon receiving new images from users, thus increasing the number of recognizable items and the classification accuracy over time. The online training is described in Section 2.3.

By trying to solve the computer vision problem of recognizing food and drink items from images, we are hoping to alleviate the issue of dietary assessment, which is why our recognition system is integrated into the PD Nutrition application for the dietary assessment of Parkinson’s disease patients [28], which is being developed in the scope of the project mHealth Platform for Parkinson’s Disease Management (PD_manager) [29]. In practice, the system works in the following way: Parkinson’s disease patients take an image of food or drink items using a smartphone camera, and our system performs recognition using deep convolutional neural networks on this image. The result is a food or drink label, which is then matched against a database of nutritional information, thus providing the patients with an automatic solution for food logging and dietary assessment.

## 2. Materials and Methods

### 2.1. Food and Drink Image Datasets

An extensive image dataset is critical for a food and drink image recognition system because it enables the learning of more general features and therefore helps combat overfitting, which is a common occurrence in machine learning, where a model describes random noise instead of learning generalizable knowledge. The goal was therefore to build a dataset that contains as many food and drink items as possible and where each item is represented with as many images as possible. Additionally, we also wanted to have images of foods and drinks that are local to the Central European region, since that would yield better results in the final application of the dietary-assessment system. This is because food and drink types vary by region, meaning that a localized image dataset offers a more accurate representation of the foods and drinks that would be recognized in practice. However, it is important to note that the entire data-preparation and model-training process, as well as the online training component are not specific to images of Central European foods and drinks; images and class labels could be provided for other foods and drinks and in other languages.

We first tried building the image recognition dataset using publicly-available images from recipe-gathering websites. This seemed appropriate since popular recipe websites have a large number of users, most of whom post not only the recipes themselves, but also images of the final product. However, this approach had two crucial drawbacks: First, the only useful labels the recipes contained were food categories (e.g., “meat dishes”, “vegetable dishes”, etc.), rather than specific dishes or drinks. The web pages for specific recipes also contained the recipe name, but since there were no naming rules or pre-defined dishes, the names were sometimes different for the same dish and very similar for different types of dishes. This meant that the recognition result would have to be a very general class, which contained very different food and drink items, making the recognition difficult. Additionally, since the results would be so general, the usefulness of such a model is questionable. Second, the resulting image dataset was too small to train a high-quality model: it contained less than 10,000 images because all of the images were taken from one recipe-gathering website. The reason why only one website was used is that food classes vary substantially from website to website, and a unified recipe image dataset was therefore impossible.

That is why we changed our approach and built the image dataset in a different way. Using existing food and drink class labels from the PD Nutrition dietary-assessment system, a web image search was performed for each food and drink class, and the resulting images were saved locally. To achieve this, we used the Google Custom Search API [30] inside a Python (Python Version 2.7.6 was used – Python is developed by the Python Software Foundation, Beaverton, OR, USA [31]) script that reads the class text labels and performs a Google image search for every label. Each class represents a food or drink item, and the script creates a folder for every class and stores its top image results. We chose to save 100 images per item; this offered a suitable balance between image quality and quantity, as too few images meant the dataset was not sufficiently large, and too many images meant saving a large number of images that do not necessarily contain the searched food or drink item, since Google image search returns the best results first. All of the images that we acquired are freely downloadable online and are labeled as either “Creative Commons Public Domain” or “Creative Commons No Derivatives”.

As a result, this recognition dataset we built had 520 food and drink image classes of 100 images each. However, due to the nature of web image searches, some results included irrelevant and low-quality images, as well as duplicate images. This meant that, in order to improve the overall dataset quality, images like that needed to be removed. This was done using a deep convolutional neural network model for image detection, by which we are referring to the process of classifying an image as either a food or drink image or as an image that contains anything else, similar to Kagaya et al. [18]. The detection model is described in Section 2.2.

**Figure 2:**
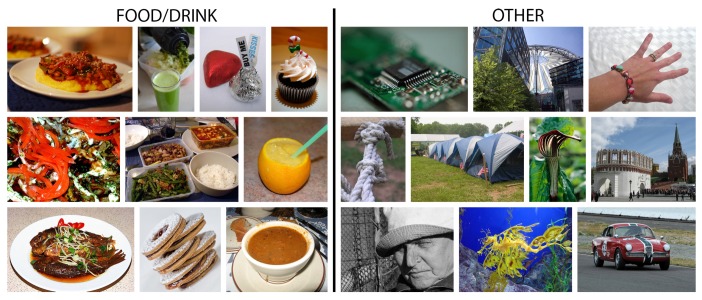
Example images from the two classes of the food and drink image detection dataset, obtained by merging recipe website images and a subset of the ImageNet dataset.

To train a model like that, a secondary image dataset needed to be built, one that contains food and drink images in one class and images of everything else in the other, which is similar to how Singla et al. structured their detection dataset [23]. This was done by merging the previously-acquired recipe image dataset, which includes images of foods, and also some drinks, and the ImageNet dataset [19]. Using another Python script, images, labeled as food or drink items, were downloaded from the ImageNet dataset, as well as a random subset of all of the other images in the dataset. The entire ImageNet dataset was not saved due to its size, which would significantly increase the training time for the food and drink image detection deep learning model and the dataset would be very unbalanced since there are many more images of other objects than there are of foods and drinks. Additionally, to further reduce the dataset imbalance and gain some rotational invariance, all of the food and drink images were rotated by 90°, 180° and 270°. These four variants per image were then saved: the resulting dataset contains 130,517 images, of which 54,564 images are food/drink images and 75,953 images contain other objects. This detection dataset is depicted in Figure 2.

The food and drink image detection model was used on the recognition dataset, and images that were labeled with “other” were removed. As the number of such images varies from class to class, the classes became unbalanced as a consequence. Like with the detection dataset, the remaining images were then rotated for a total of four variations per image, and three additional data-augmentation steps were performed on the recognition dataset to increase the dataset size and gain further invariance; the images were flipped horizontally; random color noise was introduced to them; and they were zoomed in so that 25% of all of the image borders were removed for each image. In total, there are therefore 7 such variations per image.

The dataset was then divided into training, validation and testing subsets with a 70%/10%/20% split. Additionally, two versions of the recognition dataset were created; they differ only in image size, as the images were resized to 256 × 256 pixels for the first version and to 512 × 512 pixels for the second version. The reason for having two versions of the same dataset is because our NutriNet architecture, along with the other modified architectures we tested, accepts 512 × 512 pixel images, whereas the pre-trained models accept 256 × 256 pixel images; these models and the reasoning behind using different-resolution images are described in Section 2.2. The detection dataset also contains 512 × 512 pixel images, since NutriNet was used as the final detection model. Finally, all of the datasets were transformed into the Lightning Memory-Mapped Database (LMDB) format [32] to enable a higher throughput of input images through the deep learning framework that we used, which is also described in Section 2.2.

**Figure 3:**
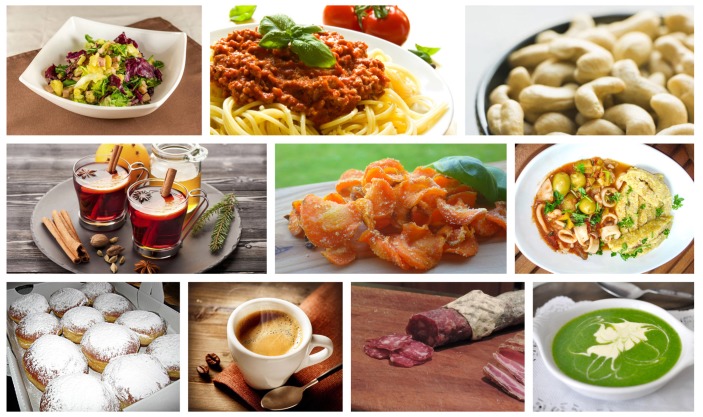
Example images from the final food and drink image recognition dataset, built from Google image searches. Each one of these images represents a different food or drink class.

Both versions of the recognition dataset contain 225,953 images of 520 different food and drink items; example images from this dataset can be seen in Figure 3. The total size of the transformed LMDB recognition dataset with larger images is 72 GB, whereas the size of the one with smaller images is 23 GB. The size of the transformed detection dataset is 46 GB. The tools needed to download images from the recognition dataset can be downloaded from the Jožef Stefan Institute website [33], and they are also available and described in the Supplementary Materials; this includes the Python script mentioned above, a complete list of all of the food and drink labels we used to create the recognition dataset and a text file with instructions on how to use the script to download the images.

### 2.2. NutriNet and Other Deep Convolutional Neural Networks

After the image datasets were acquired, we developed a food and drink image detection and recognition system that uses deep convolutional neural networks. A food or drink image is provided to the recognition model as the input, and the output is a text class label describing the food or drink item. The neural network classifies the input image into one class; if there are more food or drink items present in the image, the most prevalent one is provided as the output. For the detection model, the output is one of the two class labels: “food/drink” or “other”.

We used four different deep convolutional neural network architectures: NutriNet, which is the architecture developed in the scope of this research work, and three ImageNet Large Scale Visual Recognition Challenge (ILSVRC) winners – AlexNet (2012) [3], GoogLeNet (2014) [22] and Deep Residual Networks (ResNet, 2015) [34]. This annual challenge is one of the most important image recognition challenges, and its winners provide state-of-the-art approaches in the field. Since the challenge tasks competitors with correctly-classifying images into 1000 classes, these three architectures provided a suitable choice for training on food and drink images, as well as a comparison to the NutriNet architecture.

AlexNet is the shallowest of the three pre-existing deep neural network architectures, having five convolutional layers and three fully-connected layers. Being shallower, AlexNet learns less in-depth features, but provides faster learning times. GoogLeNet is somewhat deeper than AlexNet, having a total of 22 layers. For the purpose of this research work, we used the ResNet-152 variant of the ResNet architecture, which has 152 layers and is therefore considerably deeper than the other two ILSVRC-winning architectures. Despite this difference in layer depth, AlexNet accepts roughly the same number of parameters as ResNet, approximately 60 million, whereas GoogLeNet only accepts around four million parameters. This is due to the fact that, unlike AlexNet, GoogLeNet and ResNet do not use fully-connected layers; since these layers contribute the largest proportion of parameters, these two architectures are able to have a much higher number of layers without a considerable increase in the number of parameters. All three of them use dropout, which is a technique to prevent overfitting in neural network models by randomly excluding units in the neural network, along with their connections, during the training process [35].

Our convolutional neural network architecture, NutriNet, is a modification of the AlexNet architecture. The first difference is that while AlexNet, GoogLeNet and ResNet accept 256 × 256 pixel images and take a 227 × 227 pixel image patch (224 × 224 pixel patch for GoogLeNet and ResNet) for processing before the first layer, NutriNet accepts 512 × 512 pixel images and takes a 454 × 454 pixel image patch for processing. The reason for the difference in the input image size is that we used pre-trained models for the other deep neural network architectures; the AlexNet, GoogLeNet and ResNet models were all pre-trained on the previously-described ImageNet dataset, and these models accept 256 × 256 pixel images. On the other hand, we wanted to extract as much information from our dataset images as possible, which is why we used higher resolution images for the NutriNet architecture. Additionally, we modified the other architectures so that they accept 512 × 512 pixel images and included models using these modified architectures in the training and testing process. This was done to gain an understanding of whether a potential difference in classification accuracy between the ILSVRC-winning architectures and NutriNet is due to the higher-resolution images or due to the NutriNet architecture. All of the results are presented in Section 3. The main reason an image patch is randomly cropped before the first neural network layer is to gain some translational invariance [36].

**Figure 4:**
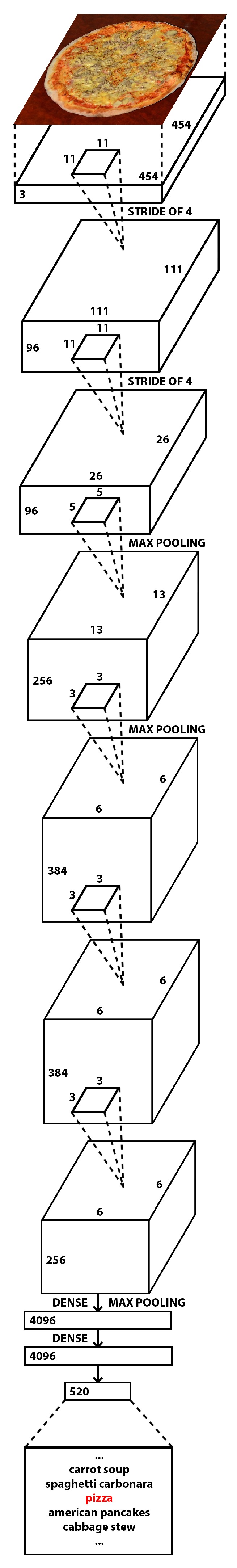
Illustration of the NutriNet architecture used on an image from the recognition dataset with a few example class labels as the output.

The second difference is that NutriNet has an additional convolutional layer at the beginning of the neural network compared to AlexNet, which means it has 6 convolutional layers in total. This convolutional layer was added to gain additional knowledge about the features in the higher resolution images. To test whether adding any further convolutional layers to the architecture would yield better results, we also tested NutriNet with an extra convolutional layer added after the input layer; we are calling this architecture NutriNet+. Lastly, as a consequence of the different input image resolutions and the additional convolutional layer, the dimensionality of the layer outputs is different. Due to this difference in dimensionality at the first fully-connected layer, NutriNet contains a considerably lower number of parameters than AlexNet: approximately 33 million. Figure 4 contains a diagram of the image classification process using the NutriNet architecture on an example image from the recognition dataset.

Using the aforementioned architectures, multiple network training parameters were defined and tested: solver type, learning rate, number of epochs and batch size. The solver type determines the method that minimizes the loss function, which is the primary quality measure in the neural network training process. The learning rate defines the rate with which the neural network’s parameters are being changed during training: higher learning rates speed up the training process, but can converge to worse loss values than lower rates. The number of epochs is the number of times all of the training images are fed through the neural network, while the batch size determines how many images are fed through at the same time. The results of this testing are presented in Section 3.

We used three different solvers: Stochastic Gradient Descent (SGD) [37], Nesterov’s Accelerated Gradient (NAG) [38] and the Adaptive Gradient algorithm (AdaGrad) [39]. All three solvers perform updates on the neural network parameters: SGD performs a parameter update for each training sample, and both NAG and AdaGrad represent upgrades to this approach. NAG computes the approximation of future parameters, thus gaining the ability to better predict local optima. AdaGrad, on the other hand, adapts the learning rate to the parameters, which means it performs larger updates for infrequent and smaller updates for frequent parameters [40].

The batch size was set according to the layer depth of the deep learning architecture that was being trained, since deeper architectures take up more space on the GPU VRAM; this way, we ensured that we used the largest possible batch size for each architecture. The idea behind this approach is that by filling the GPU VRAM with training images, we minimize the amount of data transfer between the GPU and the CPU RAM or storage drive, which decreases the amount of time the GPU is waiting for new images and thus speeds up the training process. The base learning rate was adjusted with respect to the batch size used, as per Krizhevsky [41]. For AlexNet, a batch size of 256 images and a base learning rate of 0.02 was used; for NutriNet and NutriNet+, 128 images and a rate of 0.01; for GoogLeNet, 64 images and a rate of 0.005; and for ResNet, 16 images and a rate of 0.00125. For the AlexNet, GoogLeNet and ResNet model variants accepting 512 × 512 pixel images, these values were halved in order to fit them on the GPU VRAM. Additionally, a step-down policy with a step size of 30% and *γ* = 0.1 was used for the learning rate of all of the models; these two parameters define the way and speed with which the learning rate decreases over time, with the goal of optimal loss convergence. All of the models were trained in 150 epochs and converged well before the final epoch.

Apart from using dropout, which is implemented in all of the tested deep learning architectures, another technique was used to counter overfitting: the final model was chosen at the training epoch when the loss on the validation subset stops decreasing. This signals the moment when the model stops learning image features that generalize well and instead starts overfitting on the training data. This model was then run on the testing subset once to assess its performance; the resulting accuracy values were used to compare the different deep learning architectures and solvers we tested, which is presented in Section 3.

For model training in the prototype phase, we used three tools: Caffe (NVIDIA’s fork of Caffe Version 0.15.9 was used), which is a deep learning framework developed by the Berkeley Vision and Learning Center [42]; the NVIDIA Deep Learning GPU Training System (NVIDIA DIGITS, Version 4.0 was used), which is built upon Caffe and is an interactive deep learning GPU training system that provides a graphical interface and multiple feedback options while training a model [43]; and Torch (Torch Version 7.0 was used), which is a deep learning framework, based on the Lua programming language [44]. Torch was used to train the ResNet models; as such, we used a Torch implementation of ResNet by the Facebook Artificial Intelligence Research team [45]. For all of the other models, we used a combination of Caffe and DIGITS to perform the training. The reason why Torch was used for ResNet model training is that the authors of ResNet used a modified version of Caffe to implement their deep neural network architecture [46], which means that training these models was impossible in the version of Caffe we used. For the online training of NutriNet, described in Section 2.3, only Caffe was used, since the version of the DIGITS GUI we used does not provide scheduling for automatic model training. We trained the models on GPUs because they train deep neural networks up to 13-times faster than CPUs [47]. The GPU that was used in the prototyping phase was an NVIDIA GeForce GTX TITAN X in a local computer and in the online fine-tuning phase an NVIDIA Tesla K80 in a server environment.

### 2.3. Implementing an Online Training Component

The recognition dataset that we acquired and that is described in Section 2.1 contains 520 classes of food and drink items from various food groups. Despite containing a wide variety of foods and drinks, it still represents only a small subset of all of the available food and drink items, so the aim was to develop a system that would automatically adapt and successfully recognize newly-added food and drink types.

The users, who are Parkinson’s disease patients or their carers in our case, classify a new image by taking a photograph with their smartphones. Each time this happens, the photograph is automatically uploaded and saved on our server, and the class label is then provided to the users by the recognition model. Apart from the photograph, its correct class label is also uploaded; the users have the option to correct the label provided by the deep learning model. A Python script is then run on a weekly basis to check whether there are any new images added. If there are, all new images are processed in the same way that the initial recognition dataset was processed, which is described in Section 2.1. If there is a new food or drink class among the newly-uploaded class labels, this new class is added to the dataset. Upon doing that, a Google image search is performed with the new class label as its search query, and new images belonging to this class are added from the web search to complement the user images. This is done so that a newly-added food or drink class contains as many images as possible, which helps to alleviate overfitting. The entire process of adding images from a Google image search, along with the use of the detection deep learning model to remove irrelevant search results is also described in Section 2.1. Finally, the script fine-tunes the deep learning model on this updated image dataset by adjusting the parameters in the neural network.

Caffe is used as the deep learning framework for the online training component, and it uses special “prototxt” files for the definition of both the deep learning architecture and the training parameters (solver type, number of epochs, etc.). One of these files, the one that defines the deep learning architecture for the training process, needs to be updated prior to the fine-tuning process when adding a new class. This consists of changing the number of outputs in the last neural network layer to match the new number of classes and renaming this last layer to force Caffe to relearn it. This is done automatically using the previously-mentioned Python script.

**Figure 5:**
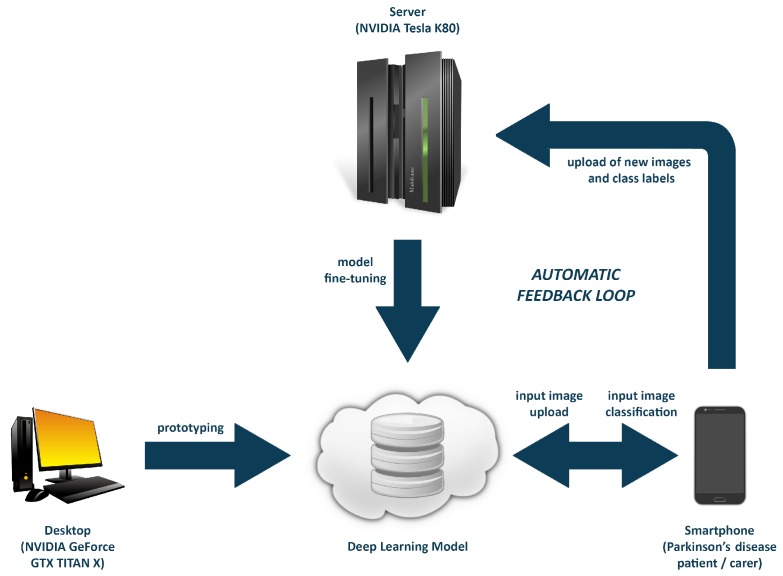
A diagram of the deep learning training process, including the online training component, which keeps the model updated.

The updated version of the model is then made available on the server to download and perform local image classification. For the purposes of PD Nutrition, the classifications of user images are performed server-side to avoid the need to re-download the model for every new version. Figure 5 illustrates the process of developing and automatically updating the deep learning model.

## 3. Results

**Figure 6:**
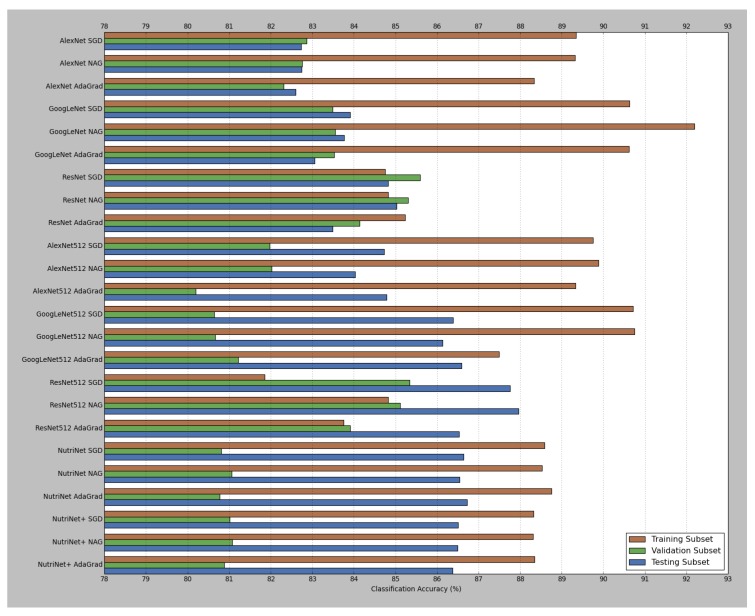
Visual representation of the classification accuracy results from Table 1. The number 512 at the end of some deep learning architecture names indicates a variant of the model that accepts 512 × 512 pixel images as input.

**Figure 7:**
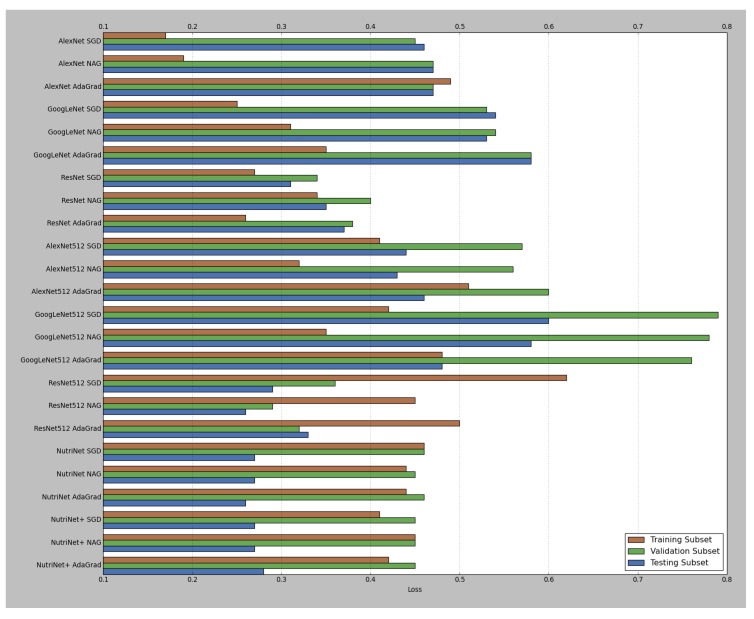
Visual representation of the loss results from Table 1. Similarly to Figure 6, the number 512 indicates a model that accepts 512 × 512 pixel images as input.

As was mentioned in Section 2.2, four different deep learning architectures (AlexNet, GoogLeNet, ResNet and NutriNet) and three solver types (SGD, NAG and AdaGrad) were tested for the recognition task; AlexNet, GoogLeNet and ResNet were tested with pre-trained models, as well as with those that accept 512 × 512 pixel images. NutriNet was additionally tested with an extra convolutional layer, and this architecture variant is called NutriNet+. Table 1 contains the results for all the tested models; classification accuracy on the testing subset of the recognition dataset (last column in Table 1) was chosen as the main quality measure for the final performance of the models. Figure 6 and Figure 7 contain a visual representation of the accuracy and loss values for all of the models. The pre-trained models with the AdaGrad solver generally performed worse than their SGD and NAG counterparts, whereas the 512 × 512 models mostly performed better with the AdaGrad solver, which seems to indicate that the learning rate selection is more important for 512 × 512 models, as AdaGrad automatically adapts the learning rate to the parameters. The ILSVRC-winning architectures achieved accuracy results according to their layer depths: the deeper the architecture, the better it performed, which is true for pre-trained, as well as 512 × 512 models. When comparing pre-trained and 512 × 512 models using the same architectures, we can see that, on average, the switch to higher-resolution images caused an increase in classification accuracy of 2.53% on the testing subset.

The best-performing model was the 512 × 512 variant of ResNet with the NAG solver, achieving a classification accuracy of 87.96%. NutriNet, on the other hand, achieved its best result with the AdaGrad solver: 86.72%, which is 1.93% higher than its AlexNet counterpart. NutriNet also achieved comparable results to GoogLeNet and was therefore outperformed only by ResNet. When comparing NutriNet to NutriNet+, we can see that the extra convolutional layer did not yield any performance increase, as NutriNet+ models achieved results that are almost identical to the results by NutriNet models. With the exception of ResNet, all models achieved their highest accuracy on the training subset. Finally, 512 × 512 models generally recorded slightly worse results on the validation subset, which is especially true for GoogLeNet, but better results on the testing subset than their pre-trained counterparts, which seems to indicate a drop in the amount of overfitting. For the detection task, NutriNet with the NAG solver was the best-performing model with a classification accuracy of 94.47%.

The training time varied from 11 to 135 h for the recognition models, depending on the deep learning architecture used; ResNet models were by far the most time consuming to train, which is due to the high layer depth of the architecture. The food and drink image detection model was trained for 19 h using the same training parameters as the NutriNet recognition models. All of the reported training times were achieved on the TITAN X GPU. While training is time consuming and computationally expensive, classifying a single image with a deep learning model takes significantly less time, making deployment possible on mobile and web applications.

### 3.1. Testing NutriNet on Other Datasets

**Figure 8:**
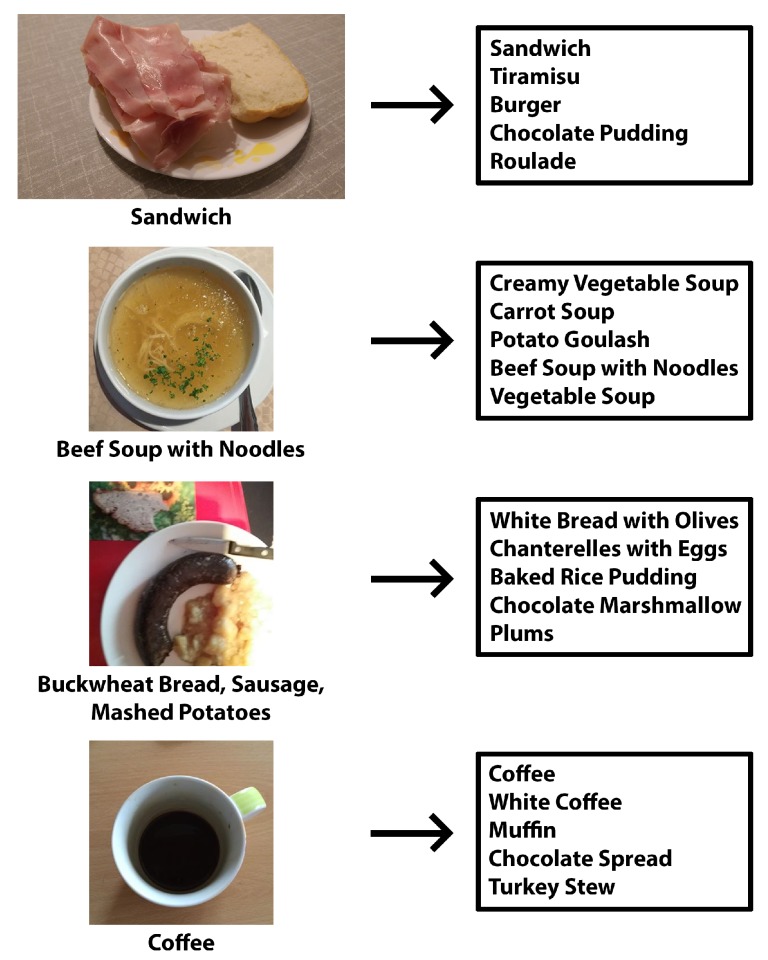
Four example images from the real-world testing dataset and their corresponding class label outputs from the NutriNet model.

To test how the trained models perform in practice, we built a small testing dataset containing real-world food and drink images. Approximately one-third of the images were taken by us, and two-thirds came from Parkinson’s disease patients. The dataset contains 200 images in total, spread across 115 of the 520 classes from our recognition dataset. For testing, we used the best-performing models for each architecture: AlexNet with the AdaGrad solver, GoogLeNet AdaGrad, ResNet NAG and NutriNet AdaGrad, all trained on 512 × 512 pixel images. AlexNet achieved a top-five accuracy of 45%, GoogLeNet 51%, ResNet 58% and NutriNet 55%. The reason we chose to measure the top-five accuracy is the way the model is used in practice: when a user classifies an image, the top five suggestions are provided and the user then chooses the correct one, which is why a top-five accuracy result is more representative of the actual recognition accuracy in practice. Figure 8 contains four distinct examples of images from this real-world dataset and their corresponding output class labels from the NutriNet model: the first image has a correct top-one classification; the second image has an incorrect top-one, but a correct top-five classification; whereas the third image has an incorrect top-five classification. The main reason this image was misclassified is that it contains three different food items, which resulted in inaccurate predictions. The real-world dataset contains more such multi-item images, which decreased the overall classification accuracy of all of the models we tested. While the first three example images contain food items, the fourth image contains a drink item with a correct top-one classification.

To further validate the results that NutriNet achieved on our datasets, we decided to test it on a publicly-available dataset. For this purpose, we chose the most recently-published one: the UNIMIB2016 food image dataset [26]. As was mentioned in Section 1.1, this dataset contains 3616 images of 73 different food items. Furthermore, since these images were collected in an Italian canteen, they contain foods that are closest to the food items present in our datasets, making UNIMIB2016 the most suitable dataset to test NutriNet on. To ensure that our results would be comparable with the baseline results, provided by the authors of the UNIMIB2016 dataset, the dataset was pre-processed as the authors suggested: food classes containing fewer than four instances were removed, leaving 65 classes, and the dataset was split into training and testing subsets. Finally, since NutriNet does not perform image segmentation, the ground-truth bounding-box information that is provided with the dataset was used to crop the food items in the dataset images. Using the NutriNet AdaGrad model, which was pre-trained on our recognition dataset, we performed fine-tuning on the UNIMIB2016 dataset, which took less than an hour. When the authors of the dataset used the ground-truth bounding boxes to segment the food images, they reported a recognition accuracy of 85.80% with their deep convolutional neural network; NutriNet outperformed this result, as it achieved an accuracy of 86.39% on the UNIMIB2016 dataset.

## 4. Discussion

The main result of our research is two-fold: the newly-defined NutriNet deep convolutional neural network architecture and the food and drink image recognition dataset, which contains a much larger number of different food types than previous efforts in the field [4,6,7,8,14,15,16,17,18,20,21,23,24,26,27] and, unlike these works, also contains a wide variety of drinks. Furthermore, since all of the images from our recognition dataset are freely available online, the dataset can be replicated by other researchers and even tailored to food and drink items local to their area. To facilitate this process, the tools we used to download images from the recognition dataset were made available online on the Jožef Stefan Institute website [33] and in the Supplementary Materials.

An additional difference between our solution and the majority of previous research is that our food and drink image recognition system is being used in practice for the dietary assessment of Parkinson’s disease patients. The accuracy results of NutriNet, presented in Section 3, are also very promising and encouraging. We achieved a classification accuracy of 86.72% for the recognition task, which is higher than the accuracy values reported by most of the other deep convolutional neural network approaches in the field [14,18,20,21,23,24,26]. The detection model achieved an accuracy of 94.47%, which is comparable to the detection results reported by other researchers [18,23]. However, since testing was performed on different datasets in these studies, the results are not directly comparable with ours. On the other hand, testing on the publicly-available UNIMIB2016 dataset showed that NutriNet outperforms the baseline method provided with the dataset [26]. Additionally, it is important to note that the classification accuracy generally decreases with the increase in the number of classes in the dataset, which makes our results even more encouraging, given that the number of classes in our recognition dataset far exceeds the solutions mentioned above.

We attribute the better results of NutriNet compared to the AlexNet deep learning architecture to the fact that it is able to gain additional knowledge from the input images as it learns a more complex representation of the input images. NutriNet achieved results on our recognition dataset that are comparable to the results by GoogLeNet, and of the tested architectures, only ResNet outperformed it. When comparing the classification accuracy of the architectures on the real-world dataset, we can observe that their order is the same as on the recognition dataset: from the lowest-performing AlexNet, to GoogLeNet and NutriNet and, finally, ResNet. However, it is important to note that NutriNet models are considerably faster to train than 512 × 512 ResNet models, with a training speed of about five epochs per hour as opposed to ResNet’s one epoch per hour (AlexNet’s and GoogLeNet’s training speeds with 512 × 512 pixel images are also slower, with 3.5 and one epoch per hour, respectively), which is mainly due to the fact that reduced image batch sizes have to be used with the deeper architectures. AlexNet, on the other hand, is slower than NutriNet because it accepts a larger number of parameters. This makes the NutriNet architecture viable for settings where training time is an important part of the problem, such as in our case, where models are continually fine-tuned on a weekly basis.

Due to the complexity of food and drink images, many of the previously-proposed methods for food recognition achieved a low classification accuracy, and drink image recognition methods were previously nonexistent. This is where deep learning comes in. Food and drink items have features that are difficult to define, making automated feature definition a more appropriate approach. The results of our research work further confirm this. However, despite using the dropout technique and selecting the model at the point in the training process when the validation loss stops decreasing, overfitting remains a problem with deep learning. In our case, the issue is that there are many different classes of food and drink items, and because the classes are unbalanced, the rarer classes generate fewer images, which introduces a greater risk of overfitting on the few images of that class that are in the dataset.

Overfitting could also be one of the reasons why the classification accuracy is lower on real-world images than on images from the testing subset, with other possible reasons being added noise and occlusion in real-world images and the fact that our recognition dataset could still contain some irrelevant images: the dataset was cleaned with a food and drink image detection model that has an accuracy of 94.47%, which means that the vast majority of images are correctly classified in the recognition dataset, but not necessarily all of them. As a consequence, this could lower the classification accuracy for real-world images. Finally, since we do not perform image segmentation, irrelevant items present in the images make the recognition task more challenging.

A shortcoming of our food and drink recognition system is that the deep learning model is limited to one output per image, which means that not every item gets successfully recognized in images with multiple food or drink items; an example of such an image is the third real-world image in Figure 8. This is true for all of the tested models and is another reason they performed worse on the real-world dataset than on the recognition dataset. In the current state of the recognition system, we are classifying 520 different food and drink items. While that number is considerably higher than in publicly-available datasets in the field [4,15,16,17,26], it is still limited when compared to the number of all of the possible foods and drinks. We address this issue by automatically adding new classes to the dataset from the class labels users provide when trying to classify a new image.

## 5. Conclusions

In this paper, we present the food and drink image detection and recognition system that we built, in the scope of which we developed a deep convolutional neural network architecture called NutriNet in order to provide a higher classification accuracy for the recognition of food and drink images from the 520-class dataset that we acquired using Google image searches, while keeping the model training time low to enable faster fine-tuning. Our recognition system is used inside the PD Nutrition dietary-assessment application for Parkinson’s disease patients, and it also incorporates online training that automatically updates the model with new images and new food and drink classes.

The next step in our research will be to further modify the NutriNet architecture, which performed well, but there is still room for improvement, especially on real-world images with added noise and obstructions. Since there are many possibilities to alter the architecture, we will be looking to implement optimization methods to automate this step, as well. As additional food and drink images are added automatically to the dataset by Parkinson’s disease patients, we also hope to further address the problem of overfitting. Additionally, to classify images with multiple food or drink items, a food and drink detection model could be trained. Each of the outputs of this model would represent a separate food or drink item that could then be used as the input to the existing recognition model. Another approach to this problem would be to join the detection and recognition steps and perform both in a single deep convolutional network; further testing would then be required to determine which of these approaches would yield better results for the final goal of food and drink image recognition.

## Figures and Tables

**Table 1:** Results of the deep learning model training on the recognition dataset. SGD, Stochastic Gradient Descent; NAG, Nesterov’s Accelerated Gradient; AdaGrad, Adaptive Gradient algorithm; ResNet, Deep Residual Networks.

Model Type	Model	Training Subset		Validation Subset		Testing Subset	
		Loss	Accuracy	Loss	Accuracy	Loss	Accuracy
Pre-Trained Models	AlexNet SGD	0.17	89.35%	0.45	82.87%	0.46	**82.73%**
	AlexNet NAG	0.19	89.32%	0.47	82.76%	0.47	**82.75%**
	AlexNet AdaGrad	0.49	88.33%	0.47	82.31%	0.47	**82.60%**
	GoogLeNet SGD	0.25	90.63%	0.53	83.49%	0.54	**83.91%**
	GoogLeNet NAG	0.31	92.19%	0.54	83.55%	0.53	**83.77%**
	GoogLeNet AdaGrad	0.35	90.62%	0.58	83.53%	0.58	**83.06%**
	ResNet SGD	0.27	84.75%	0.34	85.60%	0.31	**84.82%**
	ResNet NAG	0.34	84.82%	0.40	85.31%	0.35	**85.03%**
	ResNet AdaGrad	0.26	85.23%	0.38	84.14%	0.37	**83.49%**
512 × 512 Models	AlexNet SGD	0.41	89.76%	0.57	81.98%	0.44	**84.73%**
	AlexNet NAG	0.32	89.89%	0.56	82.03%	0.43	**84.03%**
	AlexNet AdaGrad	0.51	89.33%	0.60	80.20%	0.46	**84.79%**
	GoogLeNet SGD	0.42	90.72%	0.79	80.64%	0.60	**86.39%**
	GoogLeNet NAG	0.35	90.75%	0.78	80.66%	0.58	**86.14%**
	GoogLeNet AdaGrad	0.48	87.50%	0.76	81.22%	0.48	**86.59%**
	ResNet SGD	0.62	81.86%	0.36	85.34%	0.29	**87.76%**
	ResNet NAG	0.45	84.82%	0.29	85.11%	0.26	**87.96%**
	ResNet AdaGrad	0.50	83.76%	0.32	83.91%	0.33	**86.53%**
	NutriNet SGD	0.46	88.59%	0.46	80.81%	0.27	**86.64%**
	NutriNet NAG	0.44	88.53%	0.45	81.06%	0.27	**86.54%**
	NutriNet AdaGrad	0.44	88.76%	0.46	80.77%	0.26	**86.72%**
	NutriNet+ SGD	0.41	88.32%	0.45	81.01%	0.27	**86.51%**
	NutriNet+ NAG	0.45	88.31%	0.45	81.08%	0.27	**86.50%**
	NutriNet+ AdaGrad	0.42	88.35%	0.45	80.88%	0.28	**86.38%**

## Supplementary Materials

The tools needed to download images from the food and drink image recognition dataset detailed in this paper are available online at http://www.mdpi.com/2072-6643/9/7/657/s1 and they include the following files: “download_images.py” is a Python script used to download images from Google image search queries; “readme.txt” is a guide describing the necessary steps to obtain the food and drink image dataset using the aforementioned script; “slo_foods_drinks.txt” is a complete list of all of the food and drink class labels we used to build the recognition dataset (the list is in Slovene).
